# Jejunal gastrointestinal stromal tumor that developed in a patient with neurofibromatosis type 1: a case report

**DOI:** 10.1186/s13000-023-01398-6

**Published:** 2023-10-03

**Authors:** Hideki Nagano, Shigekazu Ohyama, Atsushi Sato, Jun Igarashi, Tomoko Yamamoto, Masumi Kadoya, Mikiko Kobayashi

**Affiliations:** 1 Department of Surgery, Marunouchi Hospital, 1-7-45, Nagisa Matsumoto, Nagano, 390-0841 Japan; 2 Department of Radiology, Marunouchi Hospital, Matsumoto Nagano, Japan; 3 Department of Pathology, Marunouchi Hospital, Matsumoto Nagano, Japan

**Keywords:** Gastrointestinal stromal tumor, Neurofibromatosis type 1, Jejunum, Neurofibromin

## Abstract

**Background:**

Neurofibromatosis type 1 (NF1) is known to be associated with the frequent occurrence of unique gastrointestinal stromal tumors (GISTs), preferably occurring in the small intestine, with no mutations in the *c-kit* proto-oncogene or platelet-derived growth factor receptor-alpha (PDGFRA), with a high tendency for multifocal development, indolent nature, with low proliferation activity and favorable prognosis.

**Case presentation:**

A woman in her forties visited her local doctor complaining of menstrual pain; a large mass was detected in her lower abdomen, and she was referred to our hospital. The patient had hundreds of skin warts and café au lait spots. The patient’s mother had been diagnosed with type 1 neurofibromatosis. The patient met the diagnostic criteria for NF1 and was diagnosed with NF1. Ultrasonography showed a large heterogeneous cystic mass with various echo patterns, solid compartments and multiple septations. Magnetic resonance imaging showed a multilocular cystic mass with liquid content exhibiting various intensities, including that of blood. A small round solid mass was also observed close to the cystic tumor. Contrast-enhanced computed tomography showed that the round solid mass showed strong enhancement in the early phase, unlike the cystic tumor component. Open laparotomy revealed a multicystic exophytic tumor measuring 11.5 cm originating from the jejunal wall, 20 cm distal to the duodenojejunal flexure. A solid tumor measuring 2.1 cm was also found on the anal side of the large tumor. We resected the short segment of the jejunum, including the two lesions. Microscopic findings revealed that the cystic and solid tumors consisted of spindle-shaped tumor cells showing little atypia with a fascicular or bundle arrangement. Nuclear mitosis was scarce. Immunostaining of the tumor cells showed positive staining for KIT and DOG1 and negative staining for S100 and desmin. The NF1 patient was diagnosed with multiple GISTs accompanied by intratumoral hemorrhagic denaturation arising from the jejunum. The TNM staging was pT4N0M0, stage IIIA.

**Conclusion:**

We report a case of GISTs associated with NF1 that showed a jejunal origin, multifocal development and few mitotic figures. The recurrence risk, survival prognosis and need for adjuvant chemotherapy, particularly in cases where the initial GIST exhibits a very indolent pathology in NF1-related GISTs, remain to be elucidated.

## Background

Neurofibromatosis type 1 (NF1), an autosomal dominant genetic disease with an incidence of 1 in 3000–4000 in the general population [1–4], is associated with the frequent occurrence of gastrointestinal stromal tumors (GISTs) with clinicopathological features distinct from those of sporadic GISTs. Whereas most sporadic GISTs occur in the stomach and possess *c-kit* proto-oncogene mutations or platelet-derived growth factor receptor-alpha (PDGFRA) mutations, GISTs associated with NF1 appear most commonly in the small intestine, especially in the jejunum, and are not accompanied by *c-kit* or PDGFRA mutations. NF1-related GISTs have also been reported to have a high tendency for multifocal development, indolent nature, low proliferation activity and comparatively favorable prognosis [5–8].

We report a case of an NF1 patient with multiple GISTs originating from the jejunum accompanied by intratumoral hemorrhagic denaturation who underwent curative resection.

This work is compliant with the SCARE checklist and has been reported in line with the SCARE criteria.

## Case report

A woman in her forties visited a local obstetrics and gynecology clinic complaining of menstrual pain. The doctor palpated a large mass in her lower abdomen, and ultrasonography revealed that the mass measured 10 cm in size, was located on the cranial side of the uterus, and reached underneath the umbilicus.

She was referred to the obstetrics and gynecology department of our hospital on the same day. Physical examination showed obesity with a body mass index of 42.3 kg/m^2^ and an abdominal mass that was difficult to palpate and not associated with tenderness. The patient had hundreds of skin warts and café au lait spots on her body. Axillary freckles were also observed. The patient reported that her mother had been diagnosed with type 1 neurofibromatosis, which was diagnosed based on café-au-lait spots and neurofibromatosis. In Japan, genetic testing for the diagnosis of neurofibromatosis is generally not performed as a matter of principle. The patient met the diagnostic criteria for NF1 and was diagnosed with NF1 upon this medical examination.

**Fig. 1:**
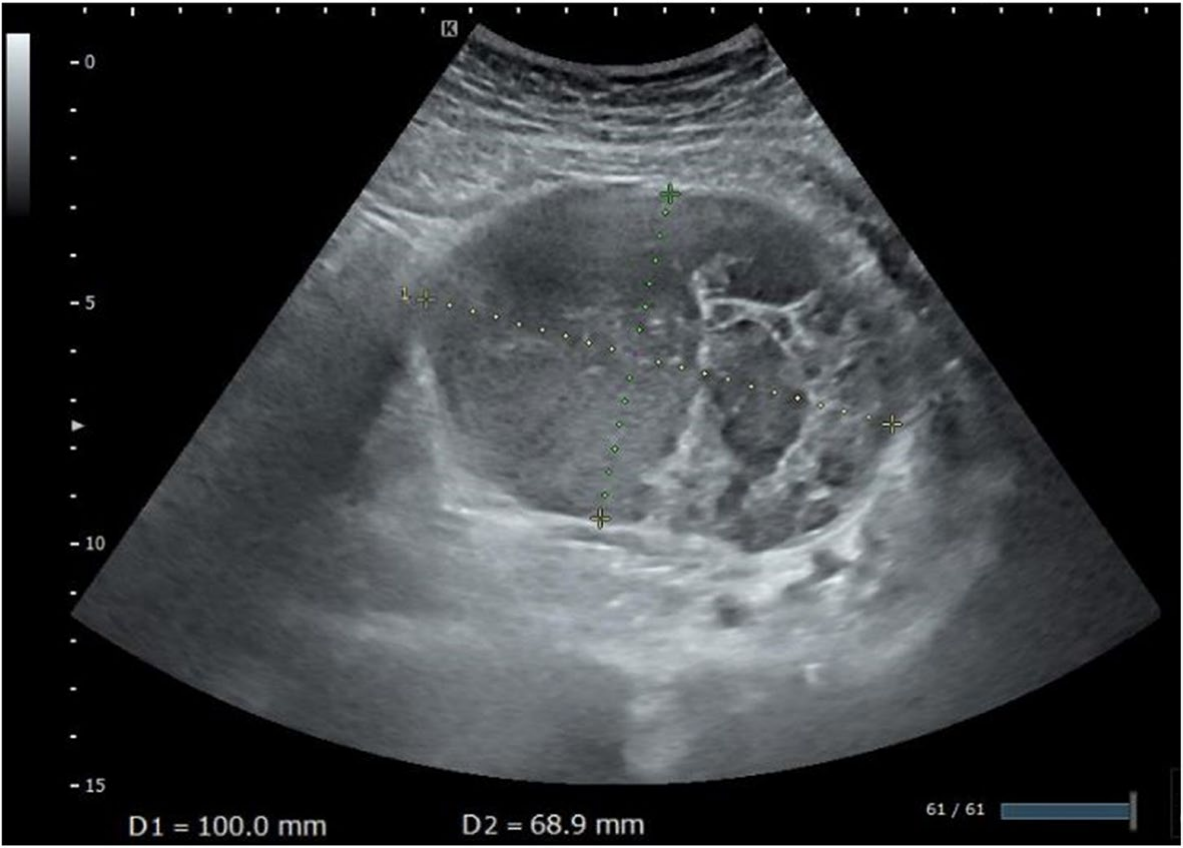
Transabdominal ultrasonography findings. A large mass in the left lower abdomen predominantly consisted of multiple cystic lesions of various sizes filled with echogenic liquid, a cavernous compartment and multiple septations and measured 10.0 × 6.9 cm

**Fig. 2:**
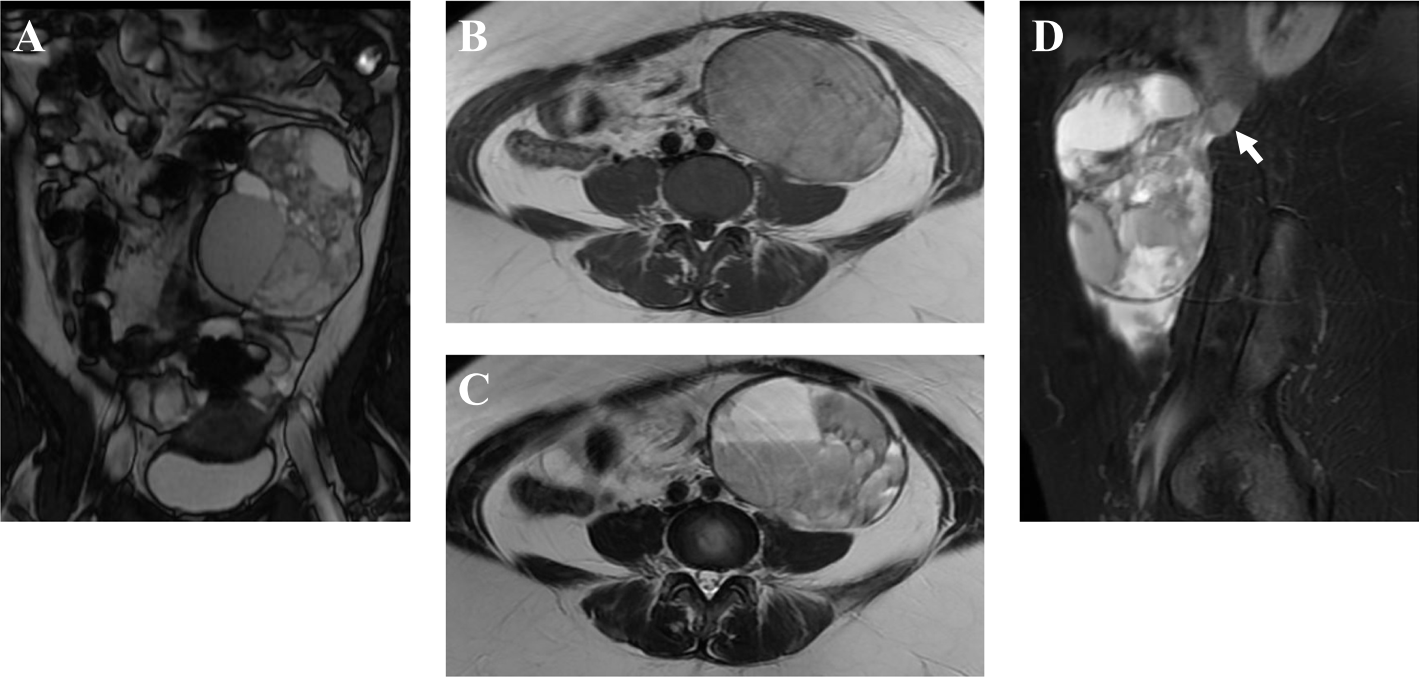
Abdominal MRI findings. **A** Coronal T2 WI with fat suppression. A multilocular cystic mass was 10.5 × 7.0 × 12.0 cm in size, and the liquid contents of the cysts showed various intensities, including that of blood. **B** T1 WI. The tumor was visualized to be iso-intense. **C** T2WI. In the largest cystic portion, the fluid fluid levels showed iso-intensity in the bottom layer and high intensity in the upper layer. **D** Sagittal T2 WI. A circumscribed homogeneous mass measuring 2.0 × 1.7 cm was also observed close to the back side of the cystic tumor

The patient’s routine hemogram and blood biochemistry parameters were within normal limits. Transabdominal ultrasonography showed a large heterogeneous predominantly cystic mass measuring 10.0 cm × 6.9 cm in the left lower abdomen that included echogenic content with a solid compartment and multiple septations (Fig. 1). Magnetic resonance imaging (MRI) showed a multilocular cystic mass measuring 10.5 × 7.0 × 12.0 cm in size in the left lower abdomen (Fig. 2). The liquid contents of the cysts exhibited various intensities, including that of blood, and the septum and solid components exhibited isointensity on both T1- and T2-weighted images (T1-WIs and T2-WIs). In the largest cystic portion, the fluid fluid levels showed isointensity in the bottom layer and high intensity in the upper layer on T2-WIs. The tumor contacted the left major psoas muscle. On the sagittal T2-WI, a circumscribed homogenous mass measuring 2.0 cm × 1.7 cm was also observed close to the back side of the cystic tumor. This tumor showed peripheral enhancement after contrast medium administration. No abnormalities were seen in the uterus or the bilateral ovary. These findings suggested a diagnosis of a mucinous cystic tumor derived from the left retroperitoneum.

**Fig. 3:**
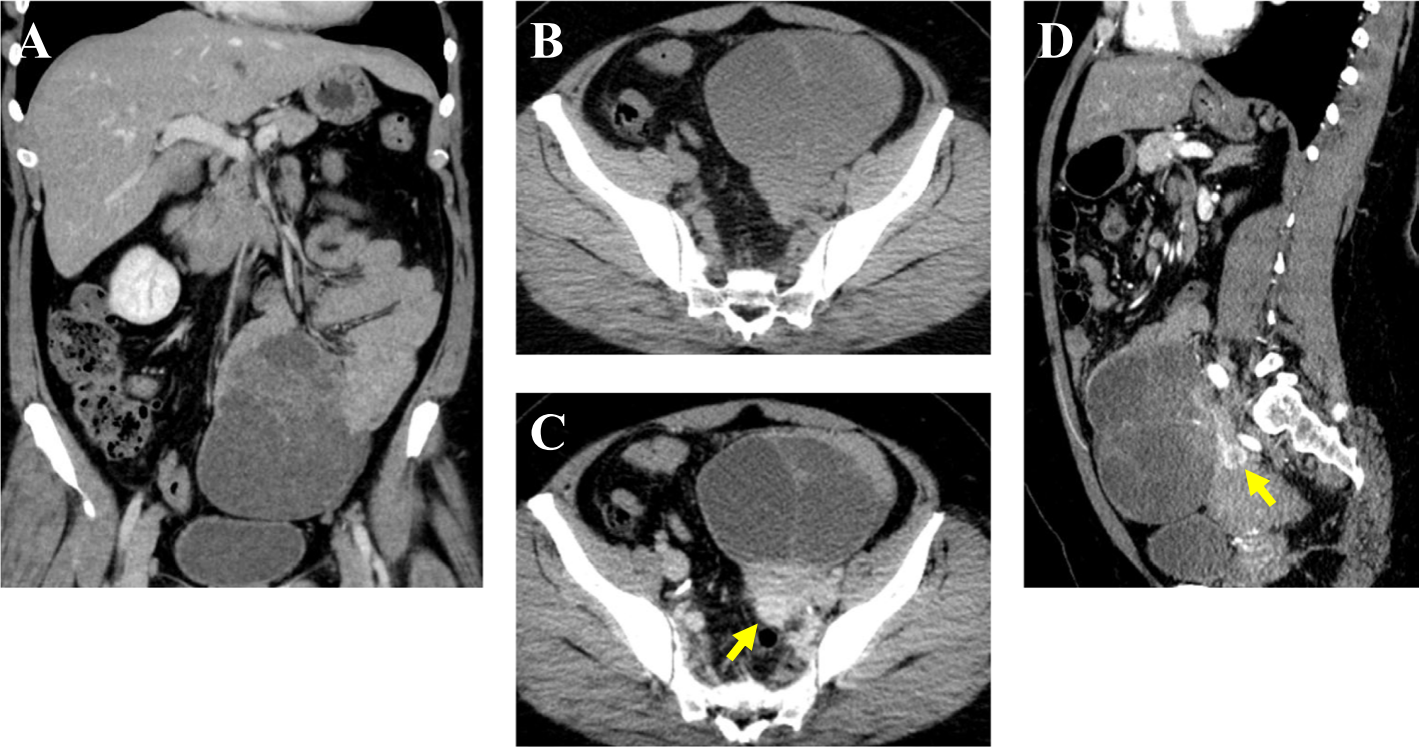
Abdominal and pelvic CT findings. **A** Coronal image. The multicystic mass changed in position to the caudal direction in comparison with the MRI findings, and a jejunal origin was suggested. No dilatation of the intestine or intraluminal component was found. **B** Plain image of the tumor. **C** Contrast-enhanced image of the tumor. The multicystic tumor showed a thin wall and multiple septations, with low-density liquid showing no enhancement and an enhanced solid compartment. The thickened portion of the left ventral region is the jejunum. On the back side of the solid compartment, there was a round solid nodular mass, measuring 18 mm in maximal diameter, showing strong enhancement in the early period of the contrast-enhanced sequence, unlike the cystic tumor component. **D** Sagittal contrast-enhanced CT image. The solid nodular mass showed a heterogeneous rim-like enhancement pattern with attenuated central areas, unlike the other cystic or solid compartments of the tumor

The patient was referred to the surgical department. Contrast-enhanced computed tomography (CT) was conducted and showed that the multicystic tumor changed in position in a caudal direction in comparison with its appearance on MRI, and a jejunal origin was suggested (Fig. 3A). The tumor had cystic changes and showed a thin wall and multiple septations, had low attenuation internally without enhancement and an enhanced solid compartment (Fig. 3B and C). A round solid nodular mass measuring 18 mm in maximal diameter was in contact with the posterior wall of the cystic tumor (Fig. 3C and D, arrow in each figure). The solid nodule showed high enhancement in the early period of the contrast-enhanced sequence, unlike the cystic tumor component. The enhancement pattern was heterogeneous and rim-like with attenuated central areas. No dilatation of the intestine or intraluminal component was found.

Open laparotomy was conducted and revealed a multicystic exophytic tumor measuring 11.5 cm in maximal diameter originating from the jejunal wall, 20 cm distal to the duodenojejunal flexure. The tumor was adherent to the abdominal wall and adjacent intestinal mesentery, and we dissected the adhesion, which also allowed us to add parietal peritoneum and fat tissue. There was no spillage or rupture of the capsule while removing the tumor. On the nearby anal side, apart from the large tumor, a solid tumor measuring 2.1 cm in size was also found. We resected short segments of the jejunum, including the two lesions, with meticulous handling and anastomosed them in a functional end-to-end manner. The recovery was uneventful, and the patient was discharged ten days after surgery.

Adjuvant chemotherapy was not administered, and follow-up examinations every 4 months with imaging were advised. She underwent follow-up CT at 4 months and 8 months after the operation, and no evidence of tumor recurrence was observed.

### Pathological findings

#### Gross pathology

**Fig. 4:**
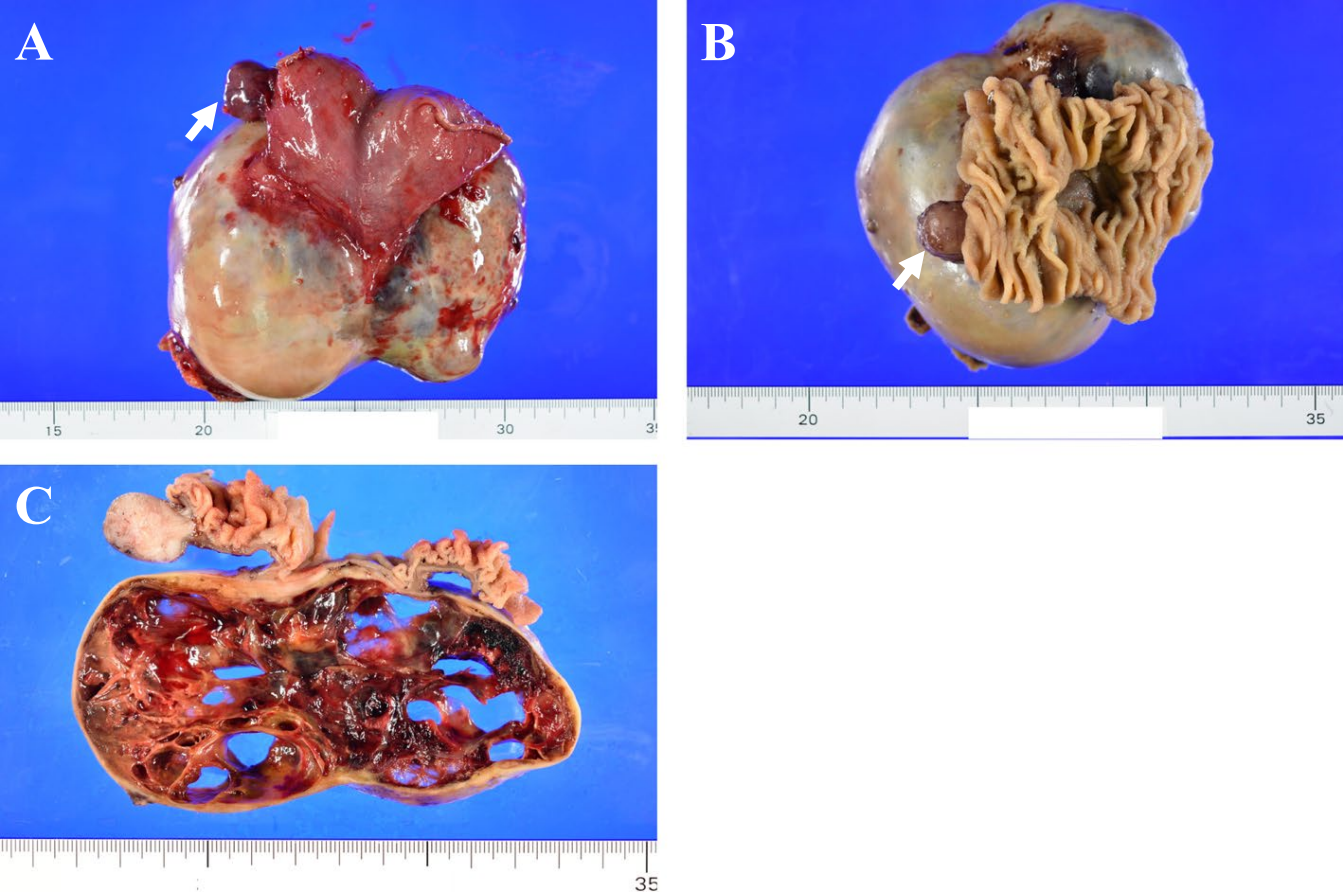
Macroscopic findings of the resected jejunal tumor. **A** Gray- and dark red-colored exophytic multicystic mass originating from the serosa aspect of the jejunum measuring 11.5 cm in maximal diameter. A small dark red solid tumor originated near the anal side of the cystic tumor measuring 2.1 cm in maximal diameter (arrow). **B** Formalin-fixed jejunal tumor. The tumor was not exposed to the mucosal aspect of the jejunum; however, there was significant thinning and shallowing of the mucosal layer in the muscle layer from which the large cystic mass developed. **C** The cut surface of the cystic tumor showed intratumoral hemorrhage and cystic degeneration and formed a multicystic lesion. Partially tan-white and myxomatous components were also observed. The small tumor showed a tan-white solid cut surface (arrow)

The gray and dark red exophytic multicystic mass originated from the serosa aspect of the jejunum, measuring 11.5 cm in maximal diameter, and the small solid tumor originated near the anal side of the cystic tumor, measuring 2.1 cm in maximal diameter (Fig. 4A). The jejunum was cut in the opposite aspect of the tumor and opened. The tumor was not exposed to the mucosal aspect; however, there was significant thinning and shallowing of the mucosal layer in the muscular layer from where the large cystic mass developed (Fig. 4B). The specimens were fixed in formalin, and then the cystic tumor and solid tumor were cut. The cut surface of the cystic tumor showed intratumoral hemorrhage and cystic degeneration, forming a multicystic lesion, and a partially tan-white and myxomatous solid component was also seen (Fig. 4C). Infiltration into the adhered abdominal wall fat was not observed. The small tumor showed a tan-white solid cut surface.

#### Microscopic findings and immunohistochemical staining

**Fig. 5:**
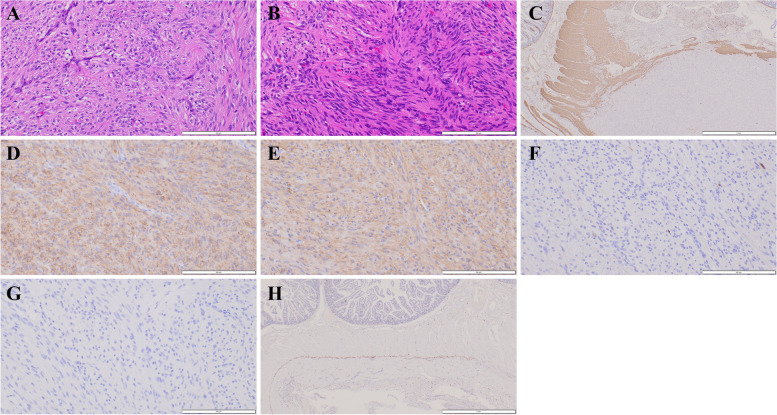
Microscopic and immunohistochemical staining findings. **A** Hematoxylin and eosin staining of the large cystic tumor. The tumor consisted of spindle-shaped tumor cells showing little atypia and forming interlacing fascicular or bundle arrangements. Nuclear mitosis was scarcely found. No lymphatic, vascular or perineural invasions were observed. The bottom right white bar indicates 200 µm. **B** Hematoxylin and eosin staining of the round solid tumor. The solid tumor showed basically similar histological features to the cystic tumor, with interlacing fascicular formations or bundle arrangements of spindle-shaped tumor cells. The bottom right white bar indicates 200 µm. **C** Immunohistochemical staining of the cystic tumor for desmin. The muscle layer of the jejunum showed positive staining for desmin. A laceration of the proper muscle layer and replacement by the tumor without invasion into the lumen side was observed, suggesting that the tumor originated from the proper muscle layer. The bottom right white bar indicates 2 mm. **D** Immunohistochemical staining for KIT in the cystic tumor. The tumor cells showed positive staining for KIT. The bottom right white bar indicates 200 µm. **E** Immunohistochemical staining for DOG1 in the cystic tumor. The tumor cells showed positive staining for DOG1. The bottom right white bar indicates 200 µm. **F** Immunohistochemical staining findings for S100. The tumor cells showed negative staining for S100. The bottom right white bar indicates 200 µm. **G** Immunohistochemical staining finding for desmin. The tumor cells showed negative staining for desmin. The bottom right white bar indicates 200 µm. **H** Immunohistochemical staining for KIT. In the proper muscle layer of the adjacent jejunum, between the inner circular muscle layer and the outside longitudinal muscle layer, KIT and DOG1 (data not shown) staining was seen in a thin linear arrangement, which was interpreted to have no association with hyperplasia of c-kit- or DOG1-positive cells, although the investigated portion of the jejunum was limited and short

The cystic tumor and the solid tumor consisted of basically similar histology, and spindle-shaped tumor cells showing little atypia formed an interlacing fascicular or bundle arrangement (Fig. 5A and B). Mixed epithelioid histology findings were not observed. The development of an abundant capillary network was also seen in the background. Nuclear mitosis was rare (1/50 high-power field in the multicystic tumor and 0/50 high-power field in the solid tumor). No lymphatic, vascular or perineural invasion was observed. A laceration of the proper muscle layer and replacement by the tumor without invasion into the lumen side was observed, suggesting that the tumor originated from the proper muscle layer (Fig. 5C). Immunohistochemically, the tumor cells showed strong pancytoplasmic staining of the type III receptor tyrosine kinase (KIT) (Fig. 5D) and the transmembrane protein discovered on GIST (DOG1) (Fig. 5E) [9] and negative staining for S100 (Fig. 5F) and desmin (Fig. 5G). In the proper muscle layer of the adjacent jejunum, between the inner circular muscle layer and the outside longitudinal muscle layer, staining for KIT and DOG1 was seen in a thin linear arrangement and was interpreted to have no association with hyperplasia of KIT-positive cells (Fig. 5H). These findings provide us with the diagnosis of multiple GISTs accompanied by intratumoral hemorrhagic denaturation arising from the jejunal proper muscle in this NF1 patient. The TNM staging (8^th^ edition of the UICC TNM classification of malignant tumors) was pT4N0M0, stage IIIA. The patient was considered high risk according to the Joensuu criteria [10] despite the scant mitosis figures because of the large size of the independent tumor (> 10 cm); the tumor site in the small intestine did not need to be included without taking the reportedly preferable prognosis of NF1-related GISTs into account.

## Discussion

Neurofibromatosis type 1 (NF1), which is also called von Recklinghausen disease, is one of the most common autosomal dominant hereditary diseases and is characterized by abnormal skin pigmentation (café au lait spots and axillary freckling), cutaneous and plexiform neurofibromas, skeletal dysplasia, and Lisch nodules (pigmented iris hamartomas), while 50% of detected mutations are reported to be de novo [1, 11, 12]. NF1 reduces average life expectancy by 15–10 years, and malignant tumors are the most common cause of death in individuals with this syndrome [1, 13]. NF1 is caused by heterozygous inactivating mutations in the NF1 gene on chromosome 17 (17q11.2), which encodes neurofibromin [1, 3, 4]. Point mutations are responsible for 90% of NF1 patients, and a single exon or whole NF1 gene deletion is associated with the remaining 5–7% [14, 15]. Neurofibromin is a member of the GTPase activating protein (GAP) family of ras regulatory proteins [1, 3, 16]. Neurofibromin can stimulate GTPase activity and negatively regulate Ras signal transduction by catalyzing the conversion of the active GTP-bound form of Ras to the inactive GDP-bound form [1, 3, 17]. In this way, neurofibromin functions as a negative regulator of the Ras/MAPK, PI3K/AKT/mTOR and Raf/MEK/ERK signaling pathways [3].

Optic pathway gliomas and neurofibromas are the most common neoplasms. Gastrointestinal abnormalities are reported in frequencies up to 5–25% in NF1 patients [6, 18]; Bakker reported the heighest frequency with neurofibromas among the gastrointestinal neoplasms except carcinoid in NF1 patients, followed by leiomyomas (13%), ganglioneurofibromas (9.8%) and GISTs (6.5%) [19]. GISTs are strongly associated with NF1, and this tumor type has been reported to have distinct characteristics, including the following: younger age of onset; development of multiple generally low-grade tumors preferably localized in the small intestine, particularly the jejunum; female predominance; and absence of KIT or PDGFRA mutations in comparison with sporadic GISTs [20, 21]. The lifetime risk of GIST occurrence for patients with NF1 has been estimated to be as high as 10–20% [22–24]. In the general population, GISTs are the most common gastrointestinal mesenchymal tumors and typically show positive staining for KIT and DOG1 immunohistochemically [9, 25]. KIT is encoded by the proto-oncogene *c-kit* [26–28], and *c-kit* gene mutations are detected in approximately 80–85% of sporadic GISTs [29]. On the other hand, NF1 patients, regardless of whether there is positive staining for KIT immunohistochemically, are known to have an absence of *c-kit* gene mutations, and the pathogenesis of GISTs among sporadic and NF1 populations is considered different; however, their distinctions in biological malignancy degree and survival prognosis remain to be elucidated [30]. Unlike sporadic GISTs accompanied by gain-of-function mutations in *c-kit* or PDGFRA, the inactivation of neurofibromin predominantly activates the MAP-kinase pathway regardless of the state of KIT expression, while the JAK-STAT3 and PI3K-AKT pathways are activated to a lesser degree in NF1-related GISTs [31].

Loss of neurofibromin activates RAS kinases and downstream kinases, including the MEK-MAPK pathway, and the subsequent expression of ETS Variant Transcription Factor 1 (ETV1), which is an E twenty-six (ETS) family transcription factor expressed in both imatinib-sensitive and imatinib-resistant GISTs required for GIST growth and survival, is a master regulator of an interstitial cell of Cajal (ICC)-GIST-specific transcription network and may induce KIT expression in NF1-associated GISTs [20, 27, 28]; this was seen in the immunostaining results in the present case. For normal ICC development and GIST survival, both KIT and ETV1 are needed, and KIT and ETV1 cooperate in GIST oncogenesis [32]. These observations might suggest that NF1 without *c-kit* mutations possesses an advantage of lower proliferation than sporadic GISTs with *c-kit* mutations due to differences in the oncogenic activity of the two tumors; this may be reflected in the reports of scant mitotic counts and favorable prognosis of NF1-related GISTs. In addition, although it is believed that patients with NF1 are born with one inactivated *NF1* allele and develop tumors when the second allele is lost [33], NF1-related GISTs have gains and losses of chromosome regions similar to those seen in sporadic GISTs, such as frequent losses of chromosomes 11, 14, 22 and 1p [31]. This might suggest that in addition to a second NF1 gene hit according to Knudson’s two-hit hypothesis [34], perhaps multiple events are needed for an NF1-related GIST to cause symptoms. Over 2,600 pathogenic gene variants leading to the onset of NF1 have been reported [3]. Since there is no clear correlation observed between these genotypes and the phenotypes, it is possible that there may be types that require changes in other genes in addition to the wild-type allele mutation. NF1-related GISTs have been reported to have an indolent nature and comparatively favorable prognosis, while a report showed that the prognosis of patients with NF1-related GISTs was similar to that of patients with conventional GISTs [21]. This dissociation has been controversial. The long-term prognosis of NF1-related GISTs has not yet been sufficiently elucidated. Specifically, it is necessary to conduct large-scale studies to determine the recurrence rate, overall survival, and need for adjuvant chemotherapy, particularly in cases where the initial GIST exhibits a very indolent pathology.

## Conclusion

We report a case of a rare subtype of GISTs associated with NF1 that shows clinical manifestations that differ from those of sporadic GISTs, such as a jejunal origin, multifocal development and scant mitotic figures. Hence, unlike sporadic GISTs, most NF1-related GISTs are not accompanied by *c-kit* mutations, and the pathogenesis of sporadic and NF1-related GISTs is considered to differ. The recurrence risk, survival prognosis and need for adjuvant chemotherapy, particularly in cases where the initial GIST exhibits a very indolent pathology in NF1-related GISTs, have not been sufficiently clarified and remain to be elucidated.

## Data Availability

All data generated or analyzed during this study are included in this published article.

## Abbreviations

NF1: Neurofibromatosis type 1
GIST: Gastrointestinal stromal tumor
PDGFRA: Platelet derived growth factor receptor-alpha
MRI: Magnetic resonance imagimg
T1-WI: T1-weighted image
T2-WI: T2-weighted image
CT: Computed tomography
KIT: Type III receptor tyrosine kinase
DOG1: Discovered on GIST
GTPase: Guanosine triphosphatase
GAP: GTPase activating protein
GTP: Guanosine triphosphate
GDP: Guanosine diphosphate
Ras: Rat sarcoma viral oncogene homolog
MAPK: Mitogen-activated protein kinase
PI3K: Phosphatidylinositol 3-kinase
mTOR: Mammalian target of rapamycin
Raf: Rapidly accelerated fibrosarcoma
MEK: Mitogen-activated extracellular signal-regulator kinase
ERK: Extracellular signal-regulated kinase
JAK: Janus kinase
STAT3: Signal transducer and activator of transcription 3
ETS: E twenty-six
ETV1: ETS Variant Transcription Factor 1
ICC: Interstitial cell of Cajal
